# Pathogenic Pathways and Therapeutic Strategies in Autosomal Dominant Polycystic Kidney Disease (ADPKD)

**DOI:** 10.33696/signaling.6.144

**Published:** 2025

**Authors:** Kenley M. Preval, Abigail O. Smith, Gregory J. Pazour

**Affiliations:** 1 Program in Molecular Medicine, University of Massachusetts Chan Medical School, 366 Plantation Street, Worcester, MA USA 01605; 2 Morningside Graduate School of Biological Sciences, University of Massachusetts Chan Medical School, 55 Lake Avenue North, Worcester MA USA 01655; 3 Current Address: Department of Pediatric Nephrology, Boston Children’s Hospital, 300 Longwood Ave, Boston MA USA 02115

**Keywords:** Cell communication and interactions, Cell signaling pathways, Polycystic kidney disease

## Abstract

Autosomal dominant polycystic kidney disease (ADPKD) is the most common inherited kidney disorder and a major cause of end-stage renal disease. The disorder is primarily caused by pathogenic variants in PKD1 or PKD2, which encode the ciliary proteins polycystin-1 and polycystin-2. Loss of polycystin function disrupts calcium and cAMP signaling within the primary cilium, altering epithelial proliferation and fluid secretion that drive cyst formation and progressive kidney enlargement. Atypical forms of ADPKD arise from variants in genes required for the production of polycystins or for ciliary assembly. Cyst growth depends on proliferative and secretory pathways involving Ca^2+^, cAMP, mTORC1, Src, and receptor tyrosine kinases, while chloride and water transport via CFTR, ANO1, and NKCC1 drive luminal expansion. The vasopressin V2 receptor antagonists tolvaptan remains the only approved therapy, but new approaches are under investigation. These include inhibitors of mTORC1, Src, and RTKs, agents that block chloride secretion, small molecules and microRNAs that restore or enhance polycystin expression, and emerging cyst-directed cytotoxic therapies. By targeting aberrant epithelial responses to disrupted polycystin function, therapeutic intervention can be developed to halt cyst initiation, expansion, and progression to renal failure.

## Autosomal Dominant Polycystic Kidney Disease

### Pathogenesis

Autosomal dominant polycystic kidney disease (ADPKD) is the most common inherited renal disorder, estimated to affect approximately 1 in 1,000 individuals worldwide [1,2]. Following diabetes and hypertension, it is the third leading cause of end-stage renal disease (ESRD) [3]. The disease is inherited in a dominant pattern, giving the offspring of affected individuals a 50% chance of acquiring the disorder. In addition, approximately 10-25% of cases do not have a family history of ADPKD, suggesting that *de novo* mutations are prevalent [4].

ADPKD presents as a systemic disorder that typically manifests during early adulthood, although cyst formation can begin *in utero* [5], and disease severity and progression vary widely among patients even with the same genetic variant. Renal manifestations include progressive cyst enlargement, reduced glomerular filtration rate (GFR), and increased total kidney volume, all contributing to kidney function decline [6,7]. The expanding cysts compress adjacent nephrons and vasculature, causing chronic kidney disease and its associated complications, such as anemia, metabolic bone disease, and elevated cardiovascular risk [8]. Other features include liver and pancreatic cysts, cardiac valvular abnormalities, intracranial aneurysms, nephrolithiasis, cyst hemorrhage, hematuria, abdominal wall hernias, and recurrent urinary tract infections [5].

Most ADPKD arises from variants in either the *PKD1* or *PKD2* gene. *PKD1*, located on chromosome 16, encodes polycystin-1, a large multidomain glycoprotein, and accounts for about 78% of cases [9,10]. *PKD2*, located on chromosome 4, encodes polycystin-2, a calcium-permeable cation channel in the transient receptor potential family, and accounts for about 15% of cases [9,11]. There are more than 1,500 unique variants in *PKD1* and *PKD2* [12]. Genetic screening can enable family risk assessment and improved patient stratification as allelic variation predicts ADPKD progression. Specifically, *PKD1* variants leading to faster ESRD onset than *PKD2* variants and truncating changes conferring greater pathogenicity than non-truncating [13]. Minor or atypical *PKD* genes are still being discovered and currently include genes like *IFT140* [14], *GANAB* [15], *ALG8* [16], and *ALG9* [17]. *GANAB, ALG8*, and *ALG9* are thought to promote the biosynthesis of polycystins [18], while IFT140 is part of the intraflagellar transport (IFT) system needed to assemble cilia [19,20].

The *ADPKD* gene products polycystin-1 and polycystin-2 are localized to primary cilia of renal epithelial cells [21,22]. Primary cilia are non-motile organelles that extend from the surface of most cells, including the epithelial cells of the kidney tubule. Defects in kidney cilia lead to cystic disease [23]. In the kidney tubule, primary cilia detect fluid shear stress or a chemical cue that reports the tubule diameter and controls the proliferation of the tubule epithelium. Kidney primary cilia, like other cilia, are microtubule-based organelles composed of 500-1,000 or more unique proteins [24]. These proteins are synthesized in the cell body and transported into cilia by IFT. During IFT, large protein complexes called IFT particles are transported along ciliary microtubules by kinesin-2 [25] and dynein-2 [26] motors. These particles are made of IFT-A, IFT-B, and BBSome subcomplexes and serve as motor adaptors, allowing a single pair of motors to transport the diverse cargos needed to build and maintain cilia. IFT140, a leading cause of atypical ADPKD, is a subunit of IFT-A. Biallelic mutations that affect the IFT system may block or partially disrupt ciliary assembly and cause cystic disease as part of a syndrome that includes skeletal dysplasia, retinal degeneration, and other organ malformations [27]. The recent finding of dominant forms of cystic disease caused by monoallelic disruptions in IFT140 suggests that haploinsufficiency or loss of the functional allele drives disease in these individuals [14,28].

In addition to IFT defects, variants that affect the ciliary transition zone at the ciliary base are a leading cause of the cystic disease nephronophthisis [29]. Nephronophthisis causes cysts at the cortical medullary boundary and the loss of kidney mass [29-31]. This cystic kidney disease is often associated with retinal degeneration in Senior-Loken syndrome and brain malformations in Joubert syndrome [32,33]. The transition zone comprises about 20 proteins that link extensively between the microtubule cytoskeleton and the ciliary membrane [29]. The transition zone functions as a selective barrier, regulating the entry and exit of proteins and signaling molecules essential for ciliary integrity [34]. The pathomechanism of NPHP is still not entirely clear but recent studies suggest an inflammatory contribution [35] and that prostaglandin E receptor agonists could potentially be beneficial as suggested by preclinical data [36].

### Cyst initiation models

The leading model of cyst formation in ADPKD is the "second hit" model, which posits that patients inherit one loss-of-function allele. Similar to the mechanism of tumor suppressors, ADPKD initiates with the stochastic loss of the second allele. This second-allele loss initiates a clonal expansion of epithelial cells, a process supported by proliferative and secretory pathways. Alternative models include the "third hit" and the "haploinsufficiency hypothesis." In the third hit model, a second hit is insufficient to initiate cyst expansion and kidney injury is needed to initiate cyst expansion after the loss of the second allele. The haploinsufficiency hypothesis suggests that a single copy of the polycystins is insufficient to maintain tubule structure and that, with time, cysts arise without the need to lose the second allele.

Support for the second-hit hypothesis comes from studies of cystic cells isolated from affected patients. The second hit hypothesis suggests that an inactivating mutation would initiate a cyst, and the cells of a cyst would derive from clonal expansion. A methylation-sensitive assay that tracked cells due to random X-inactivation [37] found strong evidence for clonal expansion within cysts. More recent attempts to test this hypothesis used genome sequencing to determine if the cells of a cyst all contain the original second hit. Several studies of isolated cysts found that many cells have the same somatic mutation, supporting the second hit hypothesis [38,39]. Furthermore, these studies found wide evidence for truncating and non-truncating somatic mutations and loss of heterozygosity at the *PKD1* or *PKD2* loci, supporting the hypothesis that ADPKD initiates with the loss of the normal allele [38,39]. Mouse models also support the second-hit hypothesis. The *Pkd2*^*−/WS25*^ mouse model carries an unstable but functional *Pkd2* allele. Over time, recombination converts the unstable allele to a loss-of-function allele, resulting in slowly progressive disease [40,41].

Animal studies strongly support the third-hit hypothesis, suggesting kidney injury modifies disease progression. In mice, polycystin loss during the early postnatal period before about day 13 leads to rapid cyst growth. In contrast, loss of the polycystins after this point leads to slow disease progression and changes the nature of the pathology. Disease initiation in the early postnatal period affects many, if not most, tubules, causing the kidney to be filled with small cysts whereas the later loss of gene function leads to focal cystic disease with a few large cysts [42]. Ischemia-reperfusion injury accelerates disease progression in *Pkd1* mutant mice by engaging impaired injury response mechanisms that promote aberrant epithelial proliferation, leading to rapid, widespread cyst formation in the affected kidney [43-45].

Haploinsufficiency in ADPKD refers to insufficient production of functional polycystins, resulting in dose-related disease severity. This idea is supported by the observation that some human cysts do not appear to have second hits when sequenced [38,39]. The idea is well supported by mouse models. Studies of an allelic series including a *Pkd2* null and the unstable *Pkd2*^*WS25*^ allele showed that cysts could form without complete loss of the polycystin-2 and that polycystin-2 expression inversely correlated with the severity of cystic phenotype [41]. A similar pattern occurs in *Pkd1*^*nl*^ mice that express reduced levels of wild-type polycystin-1 due to a splicing defect. Mice homozygous for this allele developed cystic kidney disease even though all cells still express some polycystin-1 [46]. Similarly, studies with the *Pkd1*^*RC*^ allele support haploinsufficiency. This allele, derived from a human patient, is hypomorphic, and mice homozygous for this allele develop a slow-progressing cystic phenotype [47].

### Calcium signaling in ADPKD

The primary sequence of the polycystins suggests that they regulate intracellular calcium levels [10,11]. The phenotypes caused by *PKD1* and *PKD2* variants are similar, indicating they are likely to work together. However, *PKD1* expression is higher in development [48] while *PKD2* is maintained post-development [49], and polycystin-1 was found on the cell surface [50] whereas polycystin-2 was found in the endoplasmic reticulum [51] raising questions about whether they acted together. Cryo structures clearly show that a polycystin complex composed of one polycystin-1 and three polycystin-2 subunits form [52], and the finding of both polycystins in cilia provided a common site for action [21,22].

The current hypotheses suggest that a ciliary localized polycystin-1/polycystin-2 complex monitors tubule diameter and regulates calcium influx to control proliferation. The flow hypothesis is the leading model for the mechanism of tubule diameter sensing by the kidney cilia. This hypothesis started with work in Madin-Darby canine kidney (MDCK) cells, where bending the primary cilia with suction from a micropipette increased intracellular calcium as measured using a calcium indicator. Fluid flow also elevated intracellular calcium, with calcium-sensitive fluorescence intensity rising proportionately to the flow rate [53]. Following this, Nauli and colleagues [54] reproduced the calcium influx induced by flow and demonstrated that genetic loss of *PKD1* or perturbation of polycystin-2 by antibody treatment blocked the flow-induced calcium influx. This finding has been reproduced in subsequent studies [55-57], but later work questioned the biophysics of ciliary calcium influencing cytoplasmic calcium levels. Work from Delling and colleagues showed that mouse embryonic fibroblast cilia exposed a PKD1L1-PKD2L1 channel to the environment and this channel was able to alter the ciliary calcium levels without altering the non-ciliary cytoplasmic levels [58]. These investigators re-examined whether flow could induce calcium elevations in cilia but failed to detect elevations in response to flow in kidney epithelium or other cells. They further showed that disrupting the ciliary membrane could elevate ciliary calcium, but ciliary influx was insufficient to alter cytoplasmic calcium [59]. A further challenge to the flow detection model came from *in vivo* imaging that showed that under normal conditions of flow through the tubule, cilia are fully deflected against the tubule wall [60], a condition that would be expected to promote pro-proliferative signaling. Alternatively, cilia could detect ligands that report tubule diameter. The biophysics of the mechanism has not been worked out, but polycystin-1 bound Wnt ligands induced a calcium response [61]. While the loss of cilia and the loss of polycystin-1 both result in cystic kidneys, loss of cilia in *Pkd1* mouse models reduced cysts compared to the loss of either alone, suggesting a cyst-promoting mechanism occurs in absence of polycystin-1 that requires a cilium to become effective [62].

Whether polycystins function primarily in cilia is debated, as they are also present in other cellular locations. The leading non-ciliary sites are cell-cell junctions and the endoplasmic reticulum. Polycystin-2 is particularly abundant in the endoplasmic reticulum, which may be important for cellular calcium homeostasis [63]. Regardless of the importance of non-ciliary polycystin pools, cilia are critical for maintaining kidney structure as mutations that disrupt cilia lead to cystic disease [23], and mutations that block ciliary accumulation of polycystin-2 without affecting other functional parameters cause PKD [64]. In addition, many pathogenic *PKD1* missense variants disrupt ciliary localization without apparent effects on channel properties [65].

### cAMP signaling in ADPKD

Compared with healthy kidney cells, ADPKD renal epithelial cells have elevated levels of cAMP and exhibit heightened proliferative responses to this second messenger [66-68]. The elevated cAMP is likely the result of reduced intracellular calcium which relieves inhibition of adenylyl cyclases, particularly AC5 and AC6 [66,69,70]. Consistent with this, *Pkd1* and *Ac6* double-knockout mice develop fewer and smaller cysts than *Pkd1* mutants alone and maintain improved renal function [71]. Similarly, double knockout of *Pkd2* and *Ac5* reduces hepatic cysts, suggesting that analogous ciliary cAMP mechanisms operate in cholangiocytes [72]. Evidence suggests that localized cAMP signaling is particularly important. For example, low vasopressin concentrations stimulate proliferation in ADPKD cells without a measurable increase in total intracellular cAMP [73]. Additionally, Hansen *et al.* showed that ciliary rather than cytosolic cAMP drives cystogenesis [74].

## Therapeutic Approaches for Reducing Cyst Burden in ADPKD

The conversion of a kidney tubule to a cyst will destroy the nephron or collecting duct where the cyst initiates. As the cyst enlarges, it will compress the surrounding normal tubules and vasculature, contributing to a decline in kidney function. Approaches that prevent the initiation of cysts, prevent the proliferation or cause the death of cystic cells, or prevent the expansion of cysts caused by fluid secretion could show therapeutic effects. This review outlines emerging strategies that target cyst epithelial proliferation and fluid secretion and also highlight approaches for selective cyst elimination by exploiting oxidative stress susceptibility or exploiting cyst-specific surface markers for drug delivery (Table 1).

### Targeting cyst growth through modulation of pro-proliferative signaling

Increased proliferation is thought to be a primary driver of cyst growth and numerous pathways have been identified that promote proliferation. This section discusses efforts to target these pathways to slow or stop proliferation.

#### cAMP:

As part of the kidney’s function in regulating systemic homeostasis, increased plasma osmolality, reduced blood pressure, or reduced blood volume triggers arginine vasopressin (AVP) release from the pituitary gland [75]. AVP acts through three G protein-coupled receptors: V1aR, V1bR, and V2R [76]. V2R localizes to epithelial cells in the thick ascending limb, connecting tubules and collecting ducts, where it regulates cAMP signaling to promote fluid retention [77]. In addition to its antidiuretic effect, AVP promotes proliferation in V2R-expressing renal epithelium [73,76]. Interestingly, V2R inhibition, not V1aR or V1bR, blunts AVP-induced proliferation [76]. Distal tubule and collecting duct cells express V2R and are the predominant cell types contributing to cyst formation [78]. In ADPKD, AVP levels are elevated, promoting disease progression by increasing cAMP -driven proliferation [79,80]. PCK rats lacking AVP exhibit fewer ADPKD-related phenotypes, including reduced renal cAMP, kidney weight, and cyst burden [79]. V2R inhibitor OPC31260 reduced cyst growth, renal enlargement, and epithelial proliferation in PCK rats, *Pkd2*^*−/tm1Som*^ mice, and *Pkd2*^*WS25/−*^ mice [81-83].

The studies in rodents showing that V2R was a driver of cyst growth supported the advancement of tolvaptan into clinical development [79]. The TEMPO 3:4 and REPRISE trials evaluated tolvaptan's efficacy in patients with ADPKD [84,85]. In TEMPO 3:4, tolvaptan reduced total kidney volume (TKV) growth by 49% and slowed eGFR decline by 26% over three years in early-stage disease [84]. REPRISE demonstrated a 35% reduction in eGFR decline over one year in later-stage ADPKD [85]. Adverse effects included polyuria, thirst, and hepatotoxicity [85]. TEMPO 3:4 supported FDA approval for use in patients with rapidly progressing disease, while REPRISE confirmed efficacy in more advanced stages. Limited tolerability and elevations in liver enzymes constrain the clinical use of tolvaptan. Nonetheless, V2R antagonism remains the only approved pharmacologic approach for ADPKD.

#### mTORC1:

In ADPKD mouse models, mTORC1 signaling is upregulated, promoting cell growth and proliferation, which are thought to contribute to cyst expansion [86,87]. The mTORC1 inhibitor rapamycin reduced ADPKD epithelial proliferation and improved total kidney volume, kidney-to-body weight ratios, and blood urea nitrogen in mouse models of ADPKD [86,88,89]. These findings led to clinical trials of rapamycin (Sirolimus) and Everolimus, a rapamycin derivative with better oral bioavailability and shorter half-life. The drugs had significant side effects, and neither drug showed efficacy in the trials, so the trials were discontinued. A major concern in the trials was whether the effective drug dose was high enough to inhibit mTORC1 signaling in the cystic epithelium [90-93].

Building on limitations observed with free rapamycin, nanoparticle-based formulations called mesonanoparticles have been developed to enhance kidney-specific delivery. While these particles typically accumulate in the liver and spleen, they can be targeted to the kidney proximal tubule by controlling size [94]. Particles loaded with rapamycin effectively inhibited mTORC1 signaling in the kidney but did not reduce cyst growth in a slow-onset adult model of ADPKD [95]. However, further analysis is warranted as this model developed a very mild disease, and it is unclear whether efficacy would be demonstrated in the treatment timeframe. Furthermore, the Cagg-CreER driver used for *Pkd1* deletion would have also deleted the gene in the distal tubule beyond the proximal tubule where the MNPs are enriched.

#### Receptor tyrosine-kinases:

Several receptor tyrosine kinases (RTKs) converge on shared downstream effectors to activate signaling pathways that regulate cell proliferation and fibrogenesis [96-100]. In ADPKD, aberrant proliferation drives cyst expansion, while fibrosis contributes to progressive renal dysfunction [96-98]. Under normal conditions, RTK signaling is regulated by ligand-induced degradation to prevent sustained activation [101]. *Pkd1*^*null/null*^ cells exhibit impaired RTK degradation following stimulation, resulting in persistent activation of downstream effectors such as mTORC1, leading to disease progression [101]. In five-month-old *Pkd1*^*RC/RC*^ mice, RTKs PDGFRβ and FGFR1 are elevated [102]. Nintedanib, a tyrosine kinase inhibitor FDA-approved for pulmonary fibrosis, blocks autophosphorylation of multiple growth factor receptors, including FGFR, PDGFR, and VEGFR [98,102]. *In vitro*, nintedanib reduced proliferation of human ADPKD cystic epithelial cells compared to untreated and significantly reduced cyst growth when ADPKD cells were cultured in a 3D collagen matrix [102]. *Pkd1*^*RC/RC*^ and *Pkd1*^*−/−*^ ADPKD mice treated with nintedanib exhibited significantly decreased cyst growth, TKV, blood urea nitrogen, and fibrosis [102].

#### Src:

The non-receptor tyrosine kinase c-Src transduces extracellular mitogenic stimuli to signaling networks that govern proliferation, differentiation, and survival [103]. Initially identified as an oncogene, c-Src promotes malignant proliferation [103-105]. c-Src is phosphorylated by PKA in response to elevated cAMP levels, suggesting that it may be a good target for therapeutic intervention in ADPKD [106]. Bosutinib, an FDA-approved oral Src/Bcr-Abl tyrosine kinase inhibitor for chronic myeloid leukemia, showed protective effects in *Pkhd1* [107] and *Pkd1* [108] rat and mouse models of cystic disease. A clinical trial showed reductions in total kidney volume, but Bosutinib had minimal effects on maintaining kidney function [109]. Tesevatinib, which is a kinase inhibitor that targets multiple kinases, including c-Scr, was effective in *Pkhd1* rat and *Bicc1* mouse models of cystic disease [110] and a clinical trial was undertaken, but results have not been published (clinicaltrials.gov identifier NCT01559363).

## Targeting Cyst Expansion Through Modulation of Secretion

Luminal fluid accumulation is likely to promote cyst expansion and enlarging cysts that impinge on healthy tubules likely drive renal functional decline. Fluid movement into cysts is thought to be driven by chloride transport across the apical surface via the CFTR and ANO1 transporters with cellular chloride replenished via NKCC1 basal lateral transport. This section discusses various approaches that are being explored as therapeutic options to reduce fluid movement into cysts.

Fluid accumulation in cysts is thought to result from a cAMP-facilitated movement of chloride across the apical surface which is supported by basolateral reuptake [111]. The apical chloride transporters are likely CFTR [112] and ANO1 (also known as TMEM16A) [113], while the basolateral transporter is likely the Na-K-2Cl symporter (NKCC1) [114,115]. In the kidney, CFTR is present on the tubule epithelial apical surface and retains its apical location in cystic epithelium [116]. Studies of a limited number of patients carrying CFTR and ADPKD variants found that patients with CFTR and ADPKD variants had less severe cystic disease than family members with only the ADPKD variant [117,118]. The beneficial nature of CFTR loss was supported in metanephric culture, where the loss of CFTR protected *Pkd1* mutant embryonic kidneys from cyst formation caused by 8-Br-cAMP treatment [119]. In contrast, CFTR loss was not protective against cyst formation in an early adult-onset mouse model of cystic disease driven by tamoxifen-induced knockout of *Pkd1* and *Cftr1* [120].

As discussed below, work in the CFTR field has identified small molecule effectors of CFTR activity. One compound GLPG2737 can promote the activity of the CFTR^delF508^ pathogenic variant while inhibiting wild-type CFTR in a dose-dependent manner [121]. The inhibitory effect of this drug to suppress CFTR-mediated chloride secretion has potential to be beneficial in ADPKD. Preclinical models of ADPKD demonstrated that GLPG2737 reduced cyst burden in forskolin-treated metanephric kidney explants and in both rapid and slow progression mouse models i.e., KspCreERT2-*Pkd1*^lox/lox^ and *Pkd1*^RC/RC^ mice, respectively [122]. In these models, GLPG2737 also decreased the kidney-to-body weight ratio and preserved renal function [122]. When combined with tolvaptan, GLPG2737 further suppressed cyst expansion and reduced intracellular cAMP levels [122]. Despite promising preclinical data, a randomized, double-blind, placebo-controlled Phase 2 trial (NCT04578548) evaluating this drug in ADPKD was terminated due to lack of efficacy. This outcome may reflect insufficient CFTR inhibition by the administered dose, especially since *Pkd1* loss increases CFTR expression [120]. Given its tolerability in clinical studies (NCT03410979), higher doses of GLPG2737 may be possible to achieve therapeutic CFTR suppression in ADPKD [123].

In addition to CFTR, ANO1 likely contributes to chloride-driven secretion in cyst expansion. ANO1 is a calcium-activated chloride channel upregulated in cystic epithelia of *Pkd1*^*−/−*^ mice. Genetic ablation of ANO1 reduced cyst growth in animal models. Additionally, treatment of *Pkd1*^*−/−*^ mice with ANO1 inhibitors, including niclosamide, benzbromarone, and Ani9, an ANO1-specific small molecule inhibitor, significantly slowed disease progression and decreased proliferation in renal tubular epithelial cells [113]. Currently none of these drugs have been tested in human trials. The role of ANO1 may be more complicated as ANO1 has been shown to localize to primary cilia and knockdown of expression or drug-mediated inhibition reduced cilia length [124], which could have indirect effects on cyst growth [62].

NKCC1, a basolateral Na^+^, K^+^, 2Cl^−^ cotransporter, mediates chloride reabsorption in renal epithelial cells. In ADPKD kidneys, NKCC1 and CFTR are co-expressed on some but not all cystic epithelium and function cooperatively to support chloride-driven fluid secretion [114,115,125]. Cell-based studies showed that pharmacologic inhibition of NKCC1 with bumetanide suppressed cAMP-driven cyst growth in response to forskolin and IBMX, implicating basolateral chloride influx in cyst expansion [112]. Bumetanide is being tested for benefits to chronic kidney disease (NCT03923933) but has not been explored in human trials of ADPKD.

### Targeting cyst growth through increased polycystin activity

The ADPKD-driving alleles in many patients are missense variants that could potentially retain some activity. Strategies to increase cell surface exposure of these variants have potential to reduce disease severity. Approaches include improving the biosynthesis of the polycystin variants through modulation of folding and by increasing gene expression with micro RNAs.

#### Improved folding and maturation:

Misfolded membrane proteins often accumulate in the endomembrane system during biosynthesis and fail to reach the cell surface. Drugs that improve folding and maturation of pathogenic CFTR variants have proven effective in the treatment of cystic fibrosis [126-129]. Since many ADPKD variants are likely to also affect the folding and maturation of the polycystins, this approach may also be effective in cystic disease.

The small molecule VX-809 improves folding and trafficking of CFTR, allowing it to reach the cell surface and function more effectively [130]. Evidence that VX-809 might work more generally to assist folding of membrane proteins prompted studies in ADPKD. In forskolin-induced cystic organoids, VX-809 and C18 (a compound with similar activity to VX-809) reduced cyst area and in mouse *Pkd1*^*−/−*^ models, VX-809 reduced cAMP levels and epithelial proliferation [128,131]. Another small molecule, CFTR corrector VRT-325 improved the processing, maturation, and trafficking of CFTR^delF508^ to the cell surface [130,132,133]. In mouse inner medullary collecting duct cells, treatment with VRT-325 increased ciliary levels of *Pkd1* missense variants that otherwise failed to localize to the primary cilium [65] suggesting that this approach has potential in ADPKD, but it has not been tested in clinical trial.

An alternative approach is to perturb the heat shock proteins themselves to reduce the adverse effects of misfolded proteins. One Hsp90 inhibitor, STA-2842, suppressed cyst growth, decreased kidney-to-body weight ratio, and improved renal function in *Pkd1*^*−/−*^ mice without adverse effects in wild-type mice [129] but it has not been tested in clinical trial. An alternative mechanism proposes that Hsp90 inhibition promotes primary cilia resorption and dysregulates cilia-associated proteins, a process previously shown to mitigate cystic progression caused by polycystin-1 or polycystin-2 loss [62,134].

#### Increased Expression:

MicroRNAs are short, non-coding RNAs that regulate gene expression by binding to complementary sequences on target mRNAs, leading to post-transcriptional control of protein production. Members of the miR-17~92 cluster were upregulated in *Kif3a*-knockout mouse kidneys, where the loss of this IFT kinesin II motor protein disrupts primary cilia and results in polycystic kidney disease [135,136]. Overexpression of the cluster in renal tubules increased epithelial proliferation, promoted cyst formation, and reduced expression of cyst-associated genes, including *Pkd1, Pkd2, Pkhd1*, and *Hnf1b* while kidney-specific inactivation of the cluster reduced cyst growth [136]. In addition to the miR-17~92 cluster, miR-21 is also elevated in the cyst-lining epithelium in both murine and human ADPKD kidneys. In *Pkd2*-deficient mice, genetic deletion of miR-21 reduced cyst burden, improved renal function, and extended survival. Functionally, miR-21 is thought to inhibit apoptosis in cyst-lining cells, and thus its loss enhances cell death [137].

A targeted screen within the miR-17~92 cluster identified the miR-17 family as the primary contributor to cyst growth in ADPKD as inhibition of miR-17 reduced total kidney volume, cystic burden, and epithelial proliferation in *Pkd1*^*−/−*^ and *Pkd2*^*−/−*^ ADPKD mouse models [138-140]. RGLS4326, a chemically modified antisense oligonucleotide, inhibits miR-17 activity and selectively accumulates in the kidney. Treatment with this compound increased polycystin-1 and polycystin-2 levels, reduced cyst growth in ADPKD patient-derived organoids, and improved renal function in *Pkd2*-deficient mouse models [138]. Phase1 clinical trials of RGLS4326 (NCT04536688) and the related drug RGLS8429 (NCT05429073) have recently been completed but not yet published.

### Targeting cysts with cytotoxic agents

An alternative approach to preventing cyst expansion would be to selectively kill cystic cells to prevent their proliferation. This idea was recently tested in two studies [141,142]. The first examined 11beta-dichloro, which stimulates apoptosis in cancer cells by exacerbating mitochondrial oxidative stress and causing DNA damage via alkylating activity. Treatment with 11beta-dichloro reduced cyst burden, decreased kidney volume, and improved renal function in both rapid and slow progressing mouse models. Apoptosis driven by this agent was limited to cyst-lining cells, with minimal effect on non-cystic tubule cells. Treatment also reduced fibrosis as indicated by decreased αSMA and PDGFRβ expression. A structurally related compound, 11beta-dipropyl, which lacks DNA alkylating activity, produced comparable effects, suggesting oxidative injury alone may be sufficient to induce cyst-selective cytotoxicity [141]. No clinical trials have been reported of either drug. The second study took advantage of the property of cystic epithelium to transcytose polymeric immunoglobins. An engineered dimeric monoclonal antibody directed against the cMET receptor was effectively taken up by cystic cells and reduced the levels of cMET in cyst lining cells. cMET is thought to stimulate protection against oxidative damage in cells with high metabolism. The treatment increased apoptosis of cyst lining cells and reduced the two-kidney to body weight ratio as well as the cystic index in the rapidly progressing *Bpk* mouse model of cystic disease. In a slower developing adult-onset model of *Pkd1*, treatment reduced the size of individual cysts but did not reduce the two-kidney to body weight ratio [142]. However, the mice were not followed for an extended period, and it is possible that the effects were not yet evident.

While untested *in vivo*, another approach targets cyst-lining cells with antibody-drug conjugates that carry anti-cystic drugs. This approach is being used successfully in oncology, where TACSTD2 is used as a molecular beacon to deliver cytotoxic drugs [143]. TACSTD2 (also known as TROP2) is an attractive target as its expression is generally low in most tissues but is highly upregulated in many solid tumors [144]. Two antibody-drug conjugates targeting TACSTD2 for delivery of cytotoxic drugs are FDA-approved for breast and bladder cancer [145-147]. Using RNA sequencing, we found that TACSTD2 is upregulated shortly after the deletion of *Pkd2* in a mouse model and showed elevated expression in human cyst biopsies and cystic organoids [148]. TACSTD2 overexpression in cystic epithelium supports adapting oncology-based therapeutic strategies for ADPKD. This would require the development of new antibody-drug conjugates appropriate to treat ADPKD but could potentially target cystic cells specifically to prevent cyst expansion.

## Summary

The FDA approval of tolvaptan was a major step in the development of treatment for ADPKD. However, the significant limitations of this drug make it imperative that new treatments are developed. As described in this review, the disease has a number of druggable targets and considerable energy is being expended to identify active compounds. Future work by both basic scientists and clinicians is needed to move the most promising of these drugs from the laboratory to the patient while continuing to explore the mechanism by which the polycystins regulate tubule diameter and prevent the development of cysts.

## Figures and Tables

**Figure 1.**
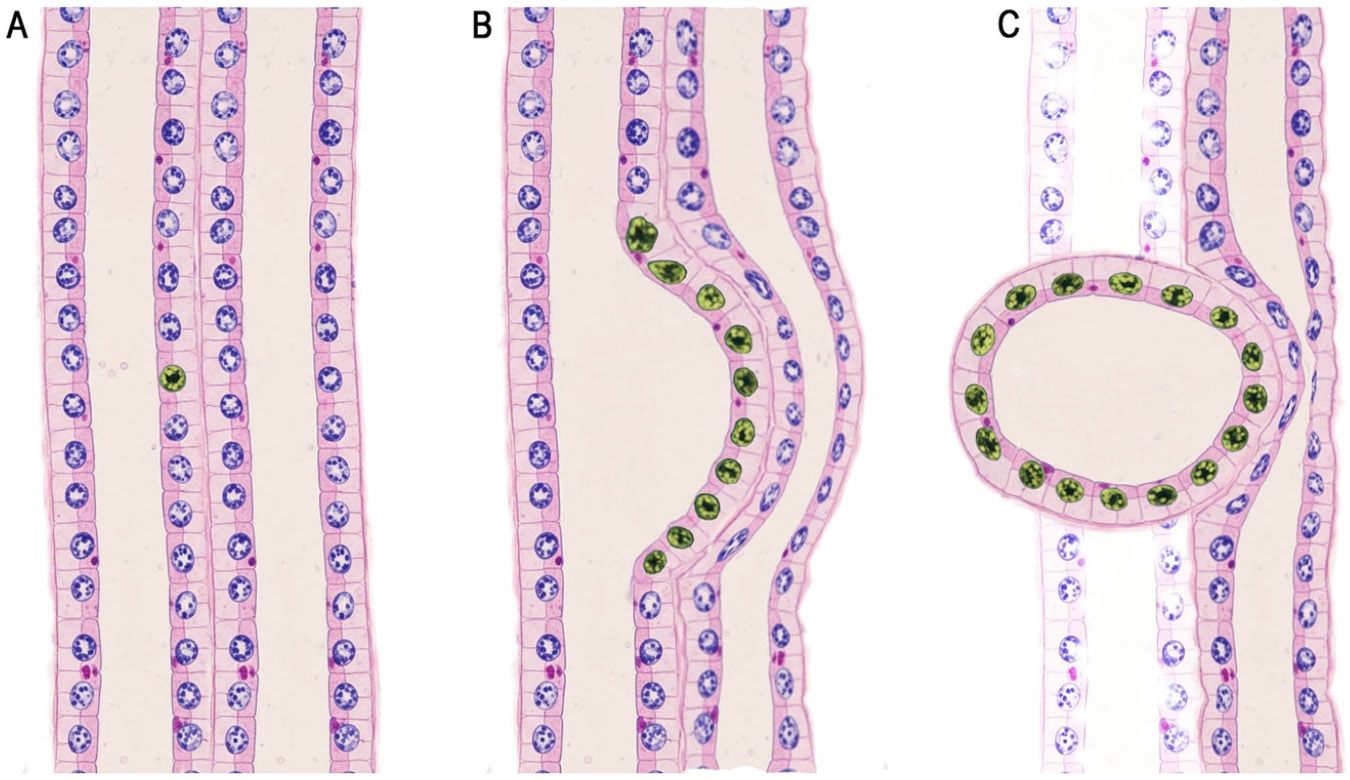
Schematic of clonal expansion and cyst initiation in nephron tubule epithelium. (**A**) The two-hit hypothesis proposes that cyst expansion is initiated by a single mutated epithelial cell within the nephron tubule. (**B**) Localized monoclonal proliferation leads to segmental tubule dilation and cyst formation. (**C**) As the cyst separates from the originating tubule, its continued growth causes mechanical compression of surrounding vasculature, triggering inflammation, fibrosis, hemorrhage, and infection. Cyst expansion contributes to tubular ischemia, obstruction, and epithelial injury, accompanied by the release of proinflammatory mediators and progressive disruption of normal tubular structure, renal architecture, and function.

**Figure 2.**
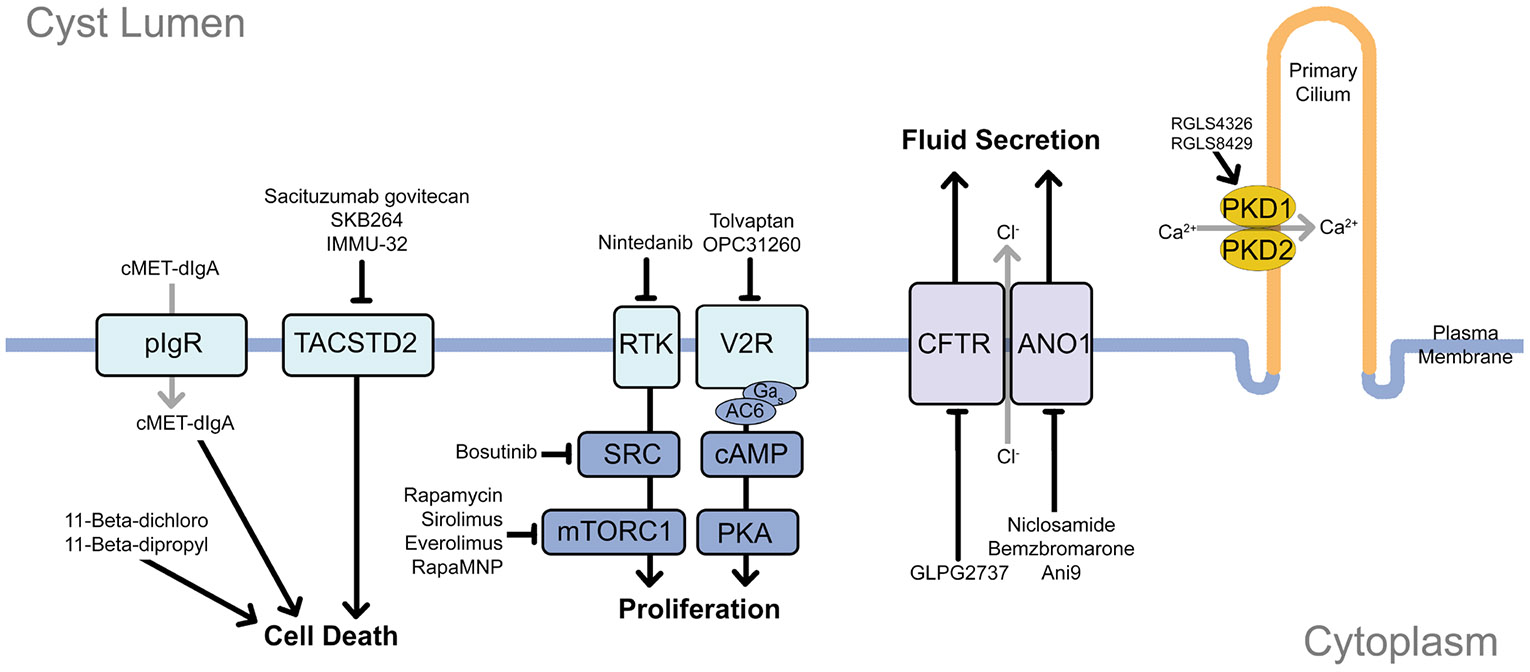
Cellular pathways and therapeutic targets in ADPKD. ADPKD epithelial cells exhibit altered signaling pathways due to gene dysregulation that works to promote cyst development and growth through proliferation and fluid secretion. RTKs activating Src and mTORC1 promote cell proliferation and growth. Their inhibition has been studied using Bosutinib, Sirolimus, Everolimus, Rapamycin, and its nanoparticle formulation, RapaMNP. Arginine vasopressin receptor 2 stimulates cAMP signaling, promoting cyst growth through proliferation. Tolvaptan and OPC31260 target this receptor. Cystic fibrosis transmembrane conductance regulator (CFTR) and anoctamin 1 (ANO1) mediate chloride secretion, enhancing cyst expansion. GLPG2737, niclosamide, benzbromarone, and Ani9 are selective inhibitors that halt cyst expansion mediated by fluid secretion. TACSTD2 is overexpressed in cystic epithelia and may be targeted with antibody-drug conjugates such as Sacituzumab govitecan, SKB264, and IMMU-132. MicroRNA-targeted therapies such as RGLS4326 and RGLS8429 promote production of polycystins. 11β-dichloro and 11β-dipropyl induce stress to promote cell death. cMET-dIgA also produces cellular stress after being transcytosed by pIgR into the lumenal space. See Table 1 for overview of targets and progress.

**Table 1.** Overview of targets and progress in clinical trials.

Target/Pathway	Drug	Status	Mechanism of Action
cAMP	Tolvaptan	FDA approved	Vasopressin V2 receptor antagonist; reduces cAMP, slowing proliferation
mTOR	Sirolimus (Rapamycin)	Clinical trial did not show efficacy	Inhibits the mTOR pathway, which controls cell growth and proliferation
	Everolimus	Clinical trial did not show efficacy	
	Rapamycin on nano particles	Mouse (Pkd1) studies were not successful	
Receptor Tyrosine Kinases	Nintedanib	Mouse (Pkd1) studies showed efficacy	Inhibits tyrosine kinases involved in cell proliferation and renal fibrosis
Non-Receptor Tyrosine Kinases	Bosutinib	Clinical trial showed reduced rates kidney enlargement but similar eGFR decline compared to placebo	Regulate proliferation, differentiation, and survival via c-Src signaling
	Tesevatinib	In clinical trial	
CFTR chloride secretion	GLPG2737	Clinical trial did not show efficacy	Inhibits wild-type CFTR chloride channel
ANO1 chloride secretion	Niclosamide	Mouse (Pkd1) studies showed efficacy	Inhibits ANO1 (TMEM16A), a calcium-activated chloride channel
	Benzbromarone	Mouse (Pkd1) studies showed efficacy	
	Ani9	Mouse (Pkd1) studies showed efficacy	
NKCC1 ion transport	Bumetanide	Cell based studies showed efficacy	Inhibits NKCC1 transporter, reducing chloride-driven fluid secretion
Polycystin Folding and Maturation	VX-809	Mouse (Pkd1) studies showed efficacy	Chaperones or correctors that improve folding and trafficking of the polycystins
	C18	Cell based studies showed efficacy	
	VRT-325	Cell based studies showed efficacy	
	STA-2842	Mouse (Pkd1) studies showed efficacy	
Increasing Polycystin Expression	RGLS4326	In clinical trial	miR-17 inhibitors; increase polycystin expression
	RGLS8429	In clinical trial	
Cytotoxic Agents	11beta-dichloro	Mouse (Pkd1) studies showed efficacy	Target cyst-lining cells for selective cytotoxicity
	11beta-dipropyl	Mouse (Pkd1) studies showed efficacy	
	Dimeric cMET antibody	Successfully reduced cyst growth in a rapidly progressing (Bpk) but not in slow developing (Pkd1) mouse models	
	TACSTD2 antibody drug conjugates	Not tested	

## Abbreviations:

ADPKD: Autosomal Dominant Polycystic Kidney Disease
AVP: Arginine Vasopressin
ESRD: End-Stage Renal Disease
GFR: Glomerular Filtration Rate
HSP: Heat Shock Proteins
IFT: Intraflagellar Transport
MNP: Mesonanoparticles
MRI: Magnetic Resonance Imaging
RTK: Receptor Tyrosine Kinase
TKV: Total Kidney Volume
